# Supplementary material for: Patterns of Intron Gain and Loss in Fungi
Source: PLoS Biol. 2004 Nov 30;2(12):e422. doi: 10.1371/journal.pbio.0020422 (PMC532390; doi:10.1371/journal.pbio.0020422)
Supplement: Table S1 — Also available at http://genes.mit.edu/NielsenEtAl/. (4.3 MB ZIP). [file pbio.0020422.st001.zip › NielsenEtAl/html/312.html]

AN0076.1.NCU03662.1.MG08303.1.FG07011.1


```
 CLUSTAL W (1.82) Multiple Sequence Alignments - Introns Inserted


Sequence 1: NCU03662.1	745 aa
Sequence 2: MG08303.1	734 aa
Sequence 3: FG07011.1	738 aa
Sequence 4: AN0076.1	726 aa
Alignment Length: 797 aa
Number Identitical Residues: 146 aa
Alignment Score (without introns) 10299


MG08303.1 	---~---------------------------------------M~PS1S~LVEDEYINGQ
NCU03662.1	---~---------------------------------------M~PV~N~-FEDPWFKGQ
FG07011.1 	MPC1CKLATVVLVVEKNFGAAELAYISLSDPVVASRAWRAQLQL~RF~F1LTEDSWINDQ
AN0076.1  	---~-------------------------------------MPI1RP~L~DDWVSGISAR
          	                                           :            :. :

MG08303.1 	AT2AHNVAELEECSIAIDATYYLQYLLHTPPTR-EPLLTALGGMN-GLDNHIREDLANWK
NCU03662.1	EV2SYNISELQECAIAVDASYFLRQLLEQAENYNEPLLTALGGLT-GIQARIQVELDNWL
FG07011.1 	AS2THDISLLEGCAIAVDATYYLCQLLETSPAH-EPLLSALGGLT-GVEAHINQNLDLWA
AN0076.1  	TS~SLPLSALKGAVVGIDASHYISQHLLHPSTR-EPLLIALGGFPFALRSNIEKELQVFK
          	   :  :: *: . :.:**::::   *  .    **** ****:  .:  .*. :*  : 

MG08303.1 	AHNVTPFFIFDGQSMMGQDEVMTSRGRKSIEKSTQAWELYVNGQAQEAVNSFGNHN~G~A
NCU03662.1	KNRTTPFFIFDGQPMVGQDEVTTKLGREIQKQTGVAWKLYSEGQAEAAVAAFGECA~S1L
FG07011.1 	KNKIVPFFVFDGQPVTGQDDITLDRGLKANKKTDEAWNLYSQGAAEEAVSTFGTSP~-~-
AN0076.1  	DLGVGCVFVFNG-LDFGKKNQRPQAHHETVRAFEHAWELYDQQQADQVVDAFSSVG1T~P
          	      .*:*:*    *:.:   .   :  .    **:** :  *: .* :*.       

MG08303.1 	FIVQNLYRMLQKILKDLDLHFLVA~PYNAAAQ0IAYLDMIDSDQ~CAGIMGPQELLLYPI
NCU03662.1	YNIQALYPMLQEELRLRKLHFLVA~PYNAAAQ~IAYLDMIDSDQ~CGGIMGPLELMLYPI
FG07011.1 	-----------------------A1PYTACA-~----------Q0CSGVMGSQELLLYPV
AN0076.1  	-RPESLYRFLQRILVQNGIDYMVA~PYSAAAQ0LAHLSTGPSPV~VDAIWGPSEVLLFDV
          	   .      .            * **.*.*.  :  .   :     .: *. *::*: :

MG08303.1 	KDMIIRSIDWTNKTATAISKRNIIKQLG--IGDNTFADALLMVGTSFLPPFPPLQDGSVL
NCU03662.1	QDCVIRSIDWTSNTCAAISKKNVMKVLN--VSEPHFIDAFLMTGTPFLPPFPPLTD-STL
FG07011.1 	KDSVLRTFDWEAKTVTAISKKKVMRSLTPTASEPRFIDSFLMAGTSFLSPFPGLLESSIY
AN0076.1  	EKLITR-IEIDPAQFFWITKQTCQEELG-RLTDEQFLDFALLLGSSFLQTFPLFENPAFP
          	:. : * ::        *:*:.  . *     :  * *  *: *:.** .** : : :  

MG08303.1 	PVPRRQNVVDAINMLRASEKSVASACHSFSDILKSHDPNWLDKYSKAKMAVAHFIYIAED
NCU03662.1	VKSQPYNIVDAINMLRTADKSVTMVSTTFHDLIKNQDPTWLDKYRKAWMAVKHFVYIAES
FG07011.1 	S---DYNISTAANLLRTAENSVATACASFNDILQNKDVDWLDKYRKARMVVHHFVYIAES
AN0076.1  	G--KAASVRDALPMFNAAGRSALALCVQYEEERRMQDLQYTDRYKRAYMNVKHHVIMDME
          	      .:  *  ::.:: .*.   .  : :  : :*  : *:* :* * * *.: :  .

MG08303.1 	GAVTVHDYDKLTKDNHEYLGLQLPAELFHYLNTGLIGPRMLSWITHSKITVLPTIDGNVS
NCU03662.1	GEVKVHDADHLTNDNHEYLGLQIPWEMFHYLNTGLIGPRLLSWITHSQVYVLPTLDGSAT
FG07011.1 	GEIRVNDYEHLTSDNHEYLGLQLPAEIFHYLNTGLIGPRLLGNITHGQVLIQPTLDGVAS
AN0076.1  	GRVGVMDAENAPTDMHEVIGQRLPEELYFYLSKGMLGPDVPNYLTSGEVLISLPLGVEDS
          	* : * * :: ..* ** :* ::* *::.**..*::** : . :* .:: :  .:.   :

MG08303.1 	DEYRNLVTKSLVPVKEMTLSLLIPRLNRGIGFRDVAMKVWFDDKFTYNVN-HR---NVQP
NCU03662.1	EEYKKLVSSQLVPLEETALSLVLARLHRGIVYKDIKAKVWYDDNYSATVLDHRHEARTKT
FG07011.1 	DEYKKLITDRIVPIKEQALSLLIPRLHRGIQHKNIKVRVWFDPKYSYTVN-HR---SINP
AN0076.1  	KIYRKVAGEILAPLREQAMFLLSNHLHRFYQTKTIRVRTWYNENADSTIT-----LRTPP
          	. *:::  . :.*:.* :: *:  :*:*    : :  :.*:: :   .:          .

MG08303.1 	SPGIRTASW-LITDSKLKSLFPKP~-ESGSIAFEVLALKNPDLVSATSTKDRIKGA~SLD
NCU03662.1	NYGVQMATWAVIKEADIKQHFPGF~-EHGSIFSEVHALTKPDFVKLTFAEQRNKAR~NVG
FG07011.1 	PPSQMVASW-DVKDDDVRAFFPED~-FASPVSLEVLSLVNTEFVDKTFPKER-PIK~GID
AN0076.1  	PVIPSIRSWKVSGDRYTEGVKKLQ0GSSGPFRFAVQSLKDSEFTPKTFASKD--TP0PLS
          	       :*    :   .       .  ...   * :* ..::.  * ...       :.

MG08303.1 	SADVIVSVAIWRFLHLRGYVSDSHELTPWGQTLAQALQAIEPVAKEHPEFEGLYEQLLVA
NCU03662.1	SASIIKSICLWRYLHVRGYIDDQHNLTNWGKALDRAMVTLEPVVKKFPTVPYLYESVLVA
FG07011.1 	STNMVTSVAIWRFLHLRGYADDKHKLTKWGNALAATLITLQDVKESNPEVTGLSEAALVA
AN0076.1  	SKDEVIANVYWQFLQLRGYINEKHQLTPWGECLEQALSVLDPE-------DSLEEATFVA
          	* . : :   *::*::*** .:.*:** **: *  :: .::           * *  :**

MG08303.1 	FEMIRFGVLHTKAPQDESHGLPLNG--~SEDDKGSLLLLSRVATLLKLRHAPTGYTGPLN
NCU03662.1	MEMVRFGLLNGSLKVVEPLSLPMHG--~DDDDKSSLMLISRCATLLKLRHQANGYTGPLS
FG07011.1 	FELIRNGLLTGKHAEGQP-GLPRKG--~SYEEKATLVLISECASLLKLRHQVYGYTGPLN
AN0076.1  	IELLRFGILNATQWFSQVSGGPMRGSV1LDEDKSFNMLISRVACIGKLRHKNIGYSGPLS
          	:*::* *:*  .    :  . * .*:    ::*.  :*:*. * : ****   **:***.

MG08303.1 	KSLLVFRSLASTVREADRDLVEAIVASMFMYAQAKRERDDSWEIGNR2LPFTQNPDIAFG
NCU03662.1	KNLLTFHSLAGEVRSAERALMEAILASLFLYGQGQRKRDDSWELSHQ2LPFLFNPDVALG
FG07011.1 	KSLLSFWSLASAVREADRDLIEAIVASMFLYGQSKRERDDQLEISRR2LPFQQEPDIGLG
AN0076.1  	RQLLSYRSLINEVRATLRNLIEITLASLFLSGDANRDRDDWTELGVS2LPFIDDNDCGLG
          	:.** : ** . ** : * *:*  :**:*: .:.:*.***  *:.   ***  : * .:*

MG08303.1 	IAVKTLLDEV--QVGEPAEVRRQRMETFPAEYVPHSTDFEEDMGIFYAFFGA~IAGAVKT
NCU03662.1	IAVKTYFDET--INWNSEKEKTERLATFPDAFVPYSMAFSEDLEIACEFFEA~VYAGLQT
FG07011.1 	IAVRTFFDDD--EAGGDKEGRLHRLEEFPKTFVPYAESLTDDFRVARDFVDA~LAKGVEA
AN0076.1  	IAVRTYLDDLPLQAEPTSQEAREEVKAKGKEWFQHSDSFTHNLERAFKLWDA0VYKGSQN
          	***:* :*: .       :   ..:      :. ::  : .::     :  * :  . : 

MG08303.1 	LPDKELSATVKGTWETATKYLELRK-------
NCU03662.1	LDGQGISANDLKAWSKAKEYLAPRILKDASGN
FG07011.1 	LGAEQMKAEDKEVWTRAQAYFAARPF------
AN0076.1  	AG---KEFKDAKIWADADKWLSDRR-------
          	      .      *  *  ::  *
```
